# Radially and Axially Oriented Ammonium Alginate Aerogels Modified with Clay/Tannic Acid and Crosslinked with Glutaraldehyde

**DOI:** 10.3390/gels10080526

**Published:** 2024-08-10

**Authors:** Lucía G. De la Cruz, Tobias Abt, Noel León, Miguel Sánchez-Soto

**Affiliations:** Centre Català del Plàstic, Universitat Politècnica de Catalunya, Barcelona Tech (EEBE-UPC), Av. d’Eduard Maristany, 16, 08019 Barcelona, Spain; tobias.abt@upc.edu (T.A.); noel.leon@upc.edu (N.L.)

**Keywords:** aerogel, freeze-drying, ammonium alginate, tannic acid, montmorillonite, crosslinking

## Abstract

Lightweight materials that combine high mechanical strength, insulation, and fire resistance are of great interest to many industries. This work explores the properties of environmentally friendly alginate aerogel composites as potential sustainable alternatives to petroleum-based materials. This study analyzes the effects of two additives (tannic acid and montmorillonite clay), the orientation that results during casting, and the crosslinking of the biopolymer with glutaraldehyde on the properties of the aerogel composites. The prepared aerogels exhibited high porosities between 90% and 97% and densities in the range of 0.059–0.191 g/cm^3^. Crosslinking increased the density and resulted in excellent performance under loading conditions. In combination with axial orientation, Young’s modulus and yield strength reached values as high as 305 MPa·cm^3^/g and 7 MPa·cm^3^/g, respectively. Moreover, the alginate-based aerogels exhibited very low thermal conductivities, ranging from 0.038 W/m·K to 0.053 W/m·K. Compared to pristine alginate, the aerogel composites’ thermal degradation rate decreased substantially, enhancing thermal stability. Although glutaraldehyde promoted combustion, the non-crosslinked aerogel composites demonstrated high fire resistance. No flame was observed in these samples under cone calorimeter radiation, and a minuscule peak of heat release of 21 kW/m^2^ was emitted as a result of their highly efficient graphitization and fire suppression. The combination of properties of these bio-based aerogels demonstrates their potential as substituents for their fossil-based counterparts.

## 1. Introduction

Reducing energy consumption is critical to achieving climate neutrality and limiting CO_2_ emissions and the greenhouse effect. Hence, thermal insulation is essential to overcome this challenge. Insulating materials, made from organic and inorganic substances, are traditionally used to control energy losses in buildings, transportation, and packaging. Synthetic foams prevent inside-out heat spreading. However, they have some drawbacks, such as pollution during production, high flammability, and non-degradability during waste disposal. Therefore, developing sustainable and minimally processed insulating materials is fundamental to mitigate such problems.

Aerogels are obtained when the solvent of a precursor gel is replaced with a gas, resulting in a highly porous material (>90%) with a high specific area. Aerogels are among the most effective thermal insulating systems because of their highly porous structure, which delays heat transport through gas and solid conduction and radiation across the pores. A micro/mesoporous, light skeleton with a minimum solid content is necessary to keep thermal conductivity at the lowest levels.

Using natural and bio-based polymers as gel precursors for aerogel development has garnered substantial research interest because of their biodegradability, availability, and non-toxicity. Bio-based aerogels derived from proteins or polysaccharides have been widely studied [1,2,3,4]. However, they tend to have drawbacks such as low mechanical properties and high flammability. Among polysaccharides, alginates have numerous carboxylic groups (-COO^−^) arranged in blocks of β-d-mannuronic (M) and α-l-guluronic (G) acid, distributed along a linear molecular chain. The ratio of M/G blocks depends on the source from which the alginate is extracted [5]. As an anionic material, alginate can form gels with bivalent and polyvalent cations, and different soluble salts can be obtained depending on the base used for its extraction (e.g., NaOH and N_4_OH). Ammonium alginate (AA) was reported to be a flame-retardant material with high NH_4_^+^ ion exchange capacity in aqueous solutions [6]. Moreover, AA can be combined with other materials to enhance their mechanical properties.

Organic–inorganic hybrid aerogels based on polymers and clay were developed and extensively investigated by Schiraldi and coworkers [7]. The rigidity of clay particles, their confinement and encapsulation by a water-soluble polymer, and their assembly into layered structures during ice growth endowed aerogels with enhanced strength and toughness. Moreover, clay also improved thermal stability and fire resistance. For instance, Chen et al. reported a 6-fold increase in mechanical resistance and a 50% reduction in the flammability of alginate aerogels when blended with an equal quantity of clay [8]. Xu et al. [9] investigated the reinforcement and fire properties of a mixture of bentonite nanoclay in ion-crosslinked sodium alginate. They reported that nanoclay served as an additional crosslinker, improving mechanical strength, elasticity, and flame self-extinction.

The orientation of polymer chains and clay particles induced during cooling plays an essential role in the thermal and mechanical properties of the aerogel. Axial freezing creates anisotropic aerogels in which the holes left by ice are axially oriented. Maximum reinforcement and thermal conductivity are expected if the aerogels are tested following the same axial direction. The opposite occurs if they are tested transversally in the freezing direction. Intermediate behavior should be expected when non-oriented freezing is applied. Recently, Guan et al. [10] compared the properties of flexible silane-crosslinked sodium alginate aerogels tested in the axial and transversal directions. They reported a 3-fold increase in the maximum load when loading coincided with the freezing direction. In addition, Zhu et al. [11] compared the mechanical and thermal properties of aerogels composed of cellulose nanofibers and calcium alginate, crosslinked with calcium ions and boric acid. They reported a minimum thermal conductivity of 30.6 mW·m^−1^·K^−1^, measured perpendicular to ice channels, but it increased to 40 mW·m^−1^·K^−1^ in the axial direction.

Tannic acid (TA) is a natural polyphenolic compound that exhibits char-forming ability during combustion. Its multiple hydroxyl groups allow its interaction with other chemical groups through hydrogen bonding or crosslinking. Thus, TA can play the double role of a flame-retardant agent and a reinforcement [12].

Therefore, this study investigated the use of alternative biomaterials for producing robust, thermal-insulating, and fire-resistant foam-like structures suitable for applications in construction or packaging. The novelty of this work relies on achieving high-performance multifunctional aerogels based on ammonium alginate through a simple preparation method. This work also explores the use of two natural modifiers (clay and TA) and a crosslinker to synergistically enhance the strength, thermal, and fire resistance of the ammonium alginate aerogel composites. Chemical crosslinking provided remarkable reinforcement, although the more compact skeleton slightly raised thermal transport and conductivity. TA and clay led to excellent fire resistance of the aerogel composites.

## 2. Results and Discussion

The samples were named based on the materials used and the freezing direction. For instance, A5C5T1-R indicates a composition of 5 g of ammonium alginate (AA), 5 g of montmorillonite (MMT), and 1 g of tannic acid (TA) added to 100 mL of deionized (DI) water, frozen in the radial direction. Samples frozen in the axial direction are denoted by X (e.g., A5C5T1-X). The crosslinked samples are differentiated with an asterisk (e.g., A5C5T1-X*) (Table S1). Orientation labels are omitted when the orientation is considered negligible for the analysis.

**Figure 1:**
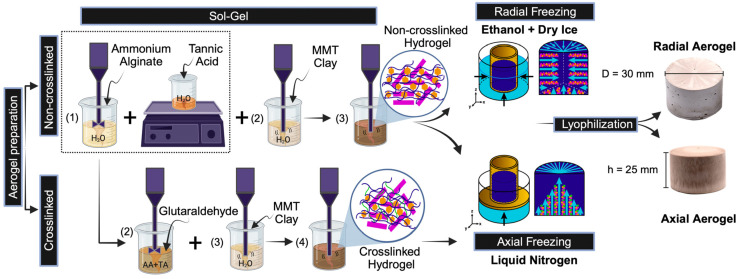
Aerogel preparation via the sol–gel method through radial and axial freeze casting (Created with Biorender.com Agrmt No. BG27599XKD).

The preparation process of the aerogel composites is depicted in Figure 1.

### 2.1. Determination of Molecular Weight

Different AA solutions at concentrations of 0.025, 0.055, and 0.090 g/100 mL were analyzed using a Ubbelohde capillary viscometer. An intrinsic viscosity of *η* = 3207.6 cm^3^/g was found using values of k = 0.0123 cm^3^/g and a = 0.960. A molecular weight (Mw) of 438,000 g/mol was determined using the Mark–Houwink–Sakurada equation (Equation (1)). This value corresponds to the higher ranges reported for alginates [13].

(1) $$\left[\eta \right]={k(Mw)}^{a}$$

### 2.2. Chemical Structure

**Figure 2:**
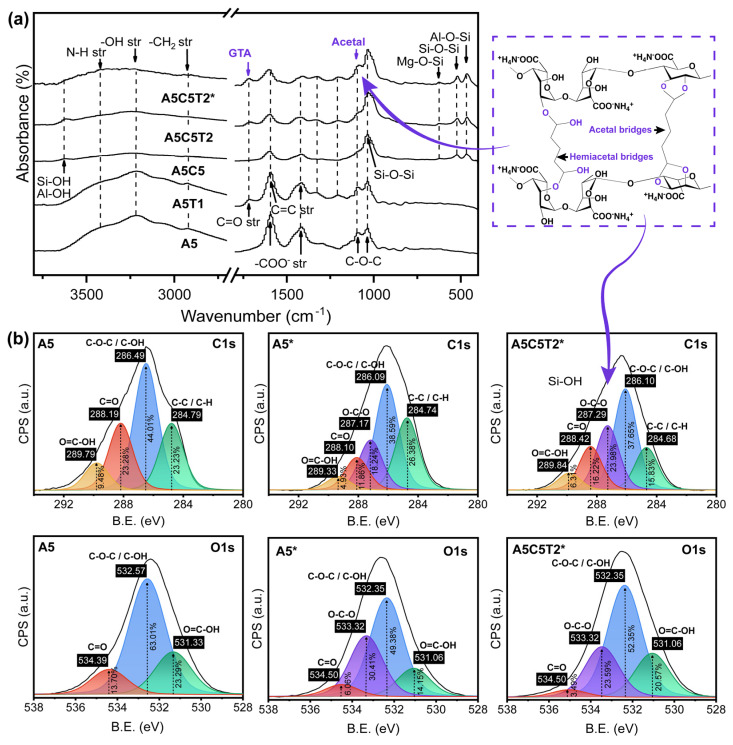
(**a**) FTIR spectra of pristine ammonium alginate (AA) aerogel before and after its modification with TA and MMT and crosslinking with GTA; (**b**) XPS spectra of non-crosslinked A5 and crosslinked A5* and A5C5T2* aerogels.

FTIR was conducted to determine the chemical interactions within the composite aerogels and highlight the main differences between the composites and the pure AA aerogel. Figure 2a presents the representative FTIR spectra of pristine AA and the effects of TA, MMT, and glutaraldehyde (GTA) addition. The proposed reactions, the main FTIR bands, and the detailed FTIR spectra of all prepared aerogels are presented in Figure S1, Table S3, and Figure S2, respectively.

The spectrum of neat AA exhibits a broad band centered at 3217 cm^−1^, characteristic of –OH stretching vibration, that overlaps with the NH stretching (asymmetry) at 3436 cm^−1^ and N-H stretching (symmetry) at 3196 cm^−1^. The band at 2920 cm^−1^ corresponds to the –CH group, whereas the intense bands at 1592 cm^−1^ and 1414 cm^−1^ belong to –COO^−^ asymmetric and symmetric vibrations, respectively [14,15]. The signal at 1089 cm^−1^ is related to the C-O of ether and ester groups, and the strong signal at 1036 cm^−1^ corresponds to C-O-C vibration from the cyclic ether bridge on the saccharide structure [16].

A slight displacement of the hydroxyl band toward a lower wavenumber (3210 cm^−1^) was detected after TA was incorporated because of the hydrogen bonding between the TA phenolic rings and the hydroxyl and carboxylic groups of alginates. A sharp peak at 1714 cm^−1^ emerged upon TA incorporation, attributed to the C=O stretching vibration of TA [17,18]. The peaks at 1592 cm^−1^ and 1416 cm^−1^ were assigned to C=C vibrations from TA benzene moieties, overlapping with the –COO^-^ signal.

New bands were observed for sample A5C5 at 3616 cm^−1^, indicative of MMT contributions from Al-OH, Si-OH, and Mg-OH vibrations in the tetrahedral and octahedral layers [19]. The decreased intensity of –COO^−^ suggests the adsorption or superficial coating of the clay, along with an increase in the peak at 1030 cm^−1^, attributed to silicon-basal oxygen vibrations (Si-O-Si) of the tetrahedral sheet [20]. Finally, the last three bands at 619 cm^−1^, 521 cm^−1^, and 463 cm^−1^ correspond to Mg-O-Si, Al-O-Si, and Si-O-Si bending vibrations, respectively [21]. The effect of TA/MMT addition can be observed in the spectrum of A5C5T2. The Al-OH and Si-OH bands increased their intensity and moved to 3610 cm^−1^, possibly related to the adsorption of TA on the MMT surface and H-bonding formation between TA and AA. 

Crosslinking with GTA formed O-C-O acetal and hemiacetal between the hydroxyl groups of TA and AA. However, O-C-O is not a proper functional group in FTIR; thus, it is detected in the ether bands (C-O-C) and is also referred to as ester and anhydride (O-C-O). The reduced intensity and broadening of the –OH and N-H bands in A5C5T2* indicate covalent linkages from acetalization. By contrast, the band located at 2920 cm^−1^, corresponding to aliphatic C-H str in GTA, and the band at 1080 cm^−1^, corresponding to the ether group from the acetal and hemiacetal linkage, increased in intensity. The latter can be attributed to the generation of covalent links between the aldehyde terminals of GTA and the hydroxyl groups of both AA and TA, resulting in a displacement of the –COO^-^ vibrations at 1600 cm^−1^ and C-O from phenolic hydroxyl vibrations at 1320 cm^−1^ and 1200 cm^−1^ [22,23]. The GTA-crosslinked FTIR spectra are shown in Figure S2b. 

The XPS elemental composition analysis was conducted on A5, A5*, and A5C5T2* to confirm the formation of acetal and hemiacetal (Figure 2b). The wide energy-scan spectra revealed the presence of C, N, O, and Na in the chemical composition of the samples (Figure S3). Table 1 shows the atomic composition and O/C ratios of the representative aerogels. As expected, the chemical structure was predominantly composed of carbon, followed by oxygen; approximately 2% of nitrogen was detected, corresponding to the NH_4_^+^ ion. The O/C atomic ratios decreased from 0.603 for A5 to 0.428 for A5* and 0.549 for A5C5T2* after GTA crosslinking. The A5* aerogel exhibited the lowest O/C atomic ratio, attributed to the loss of oxygen from hydroxyl groups after acetalization. This reduction can also be corroborated by the decrease in the –OH signal in the FTIR spectra and the increase in the C=O stretching and C-O-C bands.

The deconvolution of the C1s peaks revealed common signals in the A5 aerogel at 284.79, 286.49, 288.19, and 289.79 eV, corresponding to C-C/C-H, C-O-C/C-OH, C=O, and O-C=O, respectively [24,25]. A new signal (O-C-O) appeared at 287.17 eV, confirming the GTA crosslinking in the A5* and A5C5T2* aerogels [25,26,27]. The O1s peaks in aerogel A5 were also analyzed, revealing the presence of signals for O=C-OH at 531.33 eV, C-O-C/C-OH at 532.37 eV, and C=O at 534.39 eV. The crosslinked samples showed an overlapping curve at 533.32 eV, corresponding to O-C-O. Sample A5* presented lower intensity in the other signals compared to A5. However, the oxygen atomic percentage was higher in A5* than in A5C5T2*, indicating a higher degree of acetalization in the former as a result of the higher hydroxyl availability for the reaction with GTA and less competition with other components.

### 2.3. Density and Porosity

**Figure 3:**
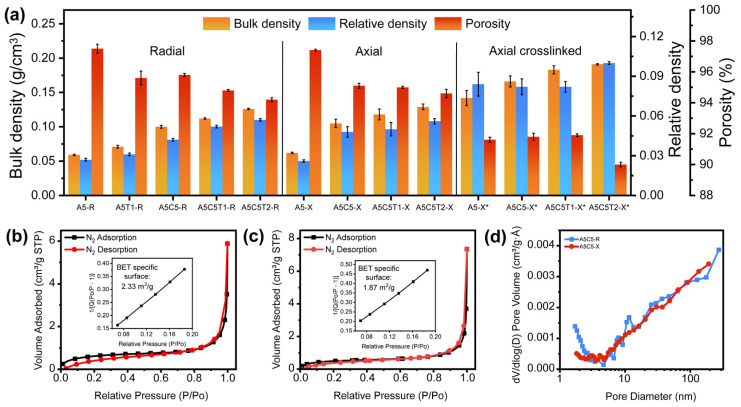
(**a**) Comparison of bulk and relative density and porosity of ammonium alginate aerogel composites: N_2_ adsorption/desorption isotherms and BET specific surface of (**b**) A5C5-R and (**c**) A5C5-X aerogels and (**d**) pore volume and pore size distribution of A5C5-R and A5C5-X aerogels.

Figure 3a illustrates the evolution of bulk densities ($\rho_{b}$), relative densities ($\rho_{r}$), and porosities for the different samples as a function of the freezing method. Table S2 presents the corresponding numerical values. Both $\rho_{b}$ and $\rho_{r}$ increased as the solid content increased; this is because MMT clay (2.6 g/cm^3^) and TA (1.56 g/cm^3^) are denser than AA (1.52 g/cm^3^). By contrast, porosity showed the opposite trend. In addition, an increase in the solid content raised the viscosity of the precursor gel, reducing the ice growth-driving force and hindering the aerogel volume expansion.

Slight variations in density were observed when comparing the radially oriented samples with their axially oriented counterparts. A 5% density increment was observed in A5-X, A5C5-X, and A5C5T1-X, and a 2% increment was observed in A5C5T2-X. These density variations were influenced by the freezing conditions. Axially oriented samples were frozen from the bottom. When the distance between the growing ice front and the cold bottom was small, the high freezing rate produced small ice crystals, resulting in narrower interlayer spacing and more compact structures [28]. The temperature gap and freezing rate decreased at greater distances from the cold side, leading to larger ice crystals and lower densities. The considerable height of the axially oriented specimens (25 mm) created a density gradient, showing small differences compared to the radially frozen specimens.

As expected, GTA crosslinking caused a further increase in $\rho_{b}$ and $\rho_{r}$ while decreasing the total porosity. The covalent bonds and short molecular chain of GTA limited ice expansion, reduced the pore size, and increased the pore wall thickness.

In addition, nitrogen adsorption/desorption isotherms were obtained for representative identical samples differing only in the radial/axial orientation to determine the effects of the freezing condition on the aerogel microstructure (Figure 3b (A5C5-R) and Figure 3c (A5C5-X)). The physisorption isotherms of the N_2_ hysteresis exhibited a reversible Type II pattern according to the IUPAC classification [29,30], indicating a macroporous structure. The BET surface area was 2.33 m^2^/g for A5C5-R and 1.87 m^2^/g for A5C5-X. Figure 3d illustrates the pore volume and pore size distribution of the selected aerogels. The mean pore size diameter ($\Phi_{p}$) was estimated considering the total pore volume obtained through $\rho_{b}$ and skeletal density ($\rho_{s}$), using the following equations:

(2) $$\Phi_{p}=\frac{4V_{p}}{S_{BET}}$$

where $V_{p}$ refers to pore volume per gram, obtained by dividing the total volume with the solid phase volume for a unitary mass, as described below:

(3) $$V_{p}=\frac{1}{\rho }-\frac{1}{\rho_{s}}$$

resulting in $\Phi_{p}\;$= 16.4 μm for A5C5-R and $\Phi_{p}\;$= 20.3 μm for A5C5-X.

### 2.4. Aerogel Morphology

**Figure 4:**
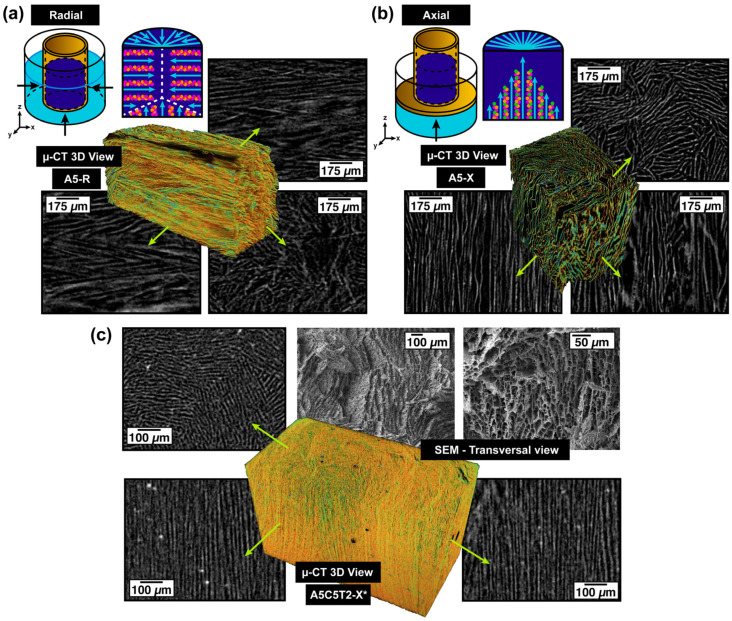
Virtual µ-CT image of pristine alginate aerogel frozen in the (**a**) radial (A5-R) and (**b**) axial (A5-X) directions and (**c**) GTA-crosslinked alginate axial aerogel composite (A5C5T2-X*).

X-ray microtomography (µ-CT) was used to investigate the 3D architecture of ice-template aerogels. The µ-CT scans depicted both the radial and axial alignments of pure alginate chains as well as the structures modified after TA/MMT/GTA addition. The 3D structure of the A5-R aerogel is shown in Figure 4a and Video S1, illustrating the predominant radial alignment resulting from the ice growing from the cylindrical molds’ walls toward its center. The white lines in the different planes represent the solid phase, whereas the black background corresponds to the voids left by ice. The structure at the bottom center of the molds is not fully oriented because of the intersection of vertical and horizontal ice-growing fronts. By contrast, the A5-X aerogel exhibited a bottom–top alignment of alginate chains (Figure 4b and Video S2).

After filler addition and crosslinking, a more compact and branched-like structure was obtained, as evidenced by the A5C5T2-X* aerogel in Figure 4c and Video S3. The longitudinal view shows a reduction in the space between the channel walls, caused by hydrogen bonding and acetal bridge formation. The top views of the A5C5T2-X* sample reveal macroporous channels arranged in clusters, corroborated by the SEM transversal section in Figure 4c.

The freezing-front velocity and the ice crystal form are the main factors governing the sample’s structure and mechanical properties. As ice crystals grow, they create a lamellar microstructure parallel to the freeze-casting direction. This effect is evident in Figure 4b,c. The axially oriented crosslinked sample exhibited a thinner lamellar microstructure caused by the temperature gradients between the bottom and the top sides of the specimen and the increment in the solid content.

**Figure 5:**
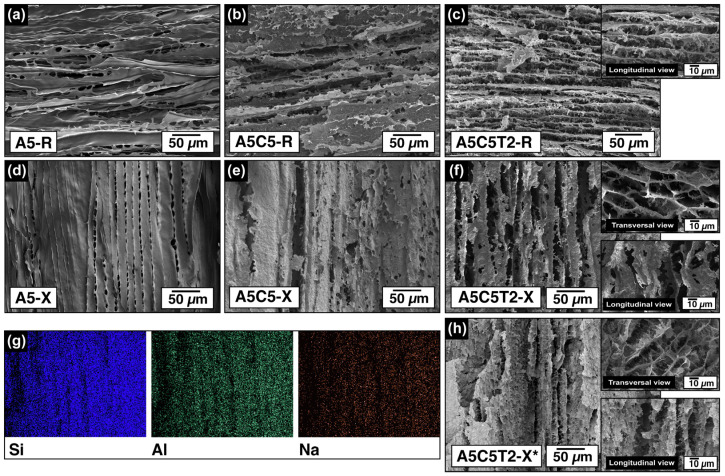
SEM images revealing the evolution of aerogel structures as the solid content increased: (**a**) A5-R, (**b**) A5C5-R, and (**c**) A5C5T2-R; pore alignment in the axial orientation: (**d**) A5-X, (**e**)A5C5-X, and (**f**) A5C5T2-X, and (**h**) after GTA crosslinking. (**g**) EDS of the dispersion of MMT clay on the A5C5T2-X* aerogel.

The SEM micrographs in Figure 5 reveal the evolution of the aerogel architecture after increasing the solid content and by the different alignments. Pristine AA frozen radially (Figure 5a) and axially (Figure 5d) displayed an oriented pore structure connected by ligaments. The A5-X sample displayed a more parallel and ordered structure than its radial counterpart, A5-R (Figure 5a), possibly due to a faster cooling rate. After adding clay, substantial structural and textural differences in both freezing directions were observed. A5C5-R (Figure 5b) and A5C5-X (Figure 5e) presented a more layered and rougher structure than A5-R and A5-X, respectively.

However, the A5C5-X sample exhibited a stronger and denser structure with more robust walls. This characteristic was attributed to the smaller freezing area, which restricts the ice growth into a more confined space. As a result, the alginate-coated clay sheets were more compactly aligned. Samples A5C5T2-R (Figure 5c) and A5C5T2-X (Figure 5f) revealed the influence of TA on the aerogel’s structure. In both cases, an increment of wall interconnections was observed, generating a dendritic/branched structure and a smaller inter-lamellar spacing because of the enhanced interaction generated by the rigid phenol rings. As longitudinal cryo-fractures revealed the ligaments that link the AA chains, MMT, and TA were analyzed in the radial direction (A5C5T2-R). Therefore, A5C5T2-X and A5C5T2-X* (Figure 5h) were transversely cryo-fractured to compare their honeycomb-like architectures with the longitudinal section of A5C5T2-R (Figure 5c).

After GTA crosslinking, A5C5T2-X* presented more struts, wall thickening, and decreased porosity. Furthermore, the energy-dispersive spectra (EDS) of the representative elements were collected to verify the homogeneous clay dispersion in the A5C5T2-X* structure (Figure 5g).

### 2.5. Compressive Behavior

Mechanical properties are crucial when considering the final aerogel applications. Low mechanical performance is common for aerogels because of their lightness. Thus, the challenge is to obtain a low-weight aerogel while enhancing its mechanical resistance.

Compression testing was performed to evaluate the mechanical properties of the different aerogels. The samples were evaluated after stabilization (Figure S4) following the cylindrical longitudinal direction. The Young’s modulus (E), yield strength (σy), and the energy absorbed (E_abs_) during deformation are listed in Table 2.

**Figure 6:**
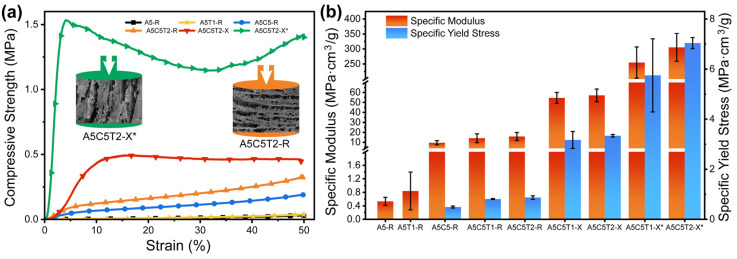
(**a**) Stress–strain compressive plots of alginate–clay–tannic acid aerogels; (**b**) specific modulus and specific yield stress of the aerogels studied.

Figure 6a depicts the comparative stress–strain curves of the aerogels, highlighting the influence of the components (A5-R to A5C5T2-R), orientation (A5C5T2-R vs. A5C5T2-X), and crosslinking (A5C5T2-X*). The simultaneous presence of clay and TA enhanced the mechanical properties beyond an increase in density (Figure 6b). The axial orientation enhanced the compressive strength. Furthermore, the crosslinked samples showed the highest mechanical properties (Table 2).

Nevertheless, adding TA did not substantially affect the Young’s modulus or yield stress of the samples, except for A5T1. By contrast, adding clay (A5C5-R) led to an 18-fold increase in these values compared to the pure AA aerogel. This notable enhancement was attributed to several factors: the high rigidity of clay, the alignment of clay platelets, and the electrostatic interactions between alginate and clay (Figure 6b).

Similarly, the A5C5T2-X sample was much more rigid (E_sp_ = 57.1 ± 6.4 MPa·cm^3^/g) than the equivalent radially oriented sample (A5C5T2-R; E_sp_ = 15.8 ± 4.0 MPa·cm^3^/g). Furthermore, when the internal walls were oriented in the same direction as the applied force, the compression resistance (σy_sp_) and the E_abs_ increased 3.8-fold and 2.6-fold, respectively.

GTA crosslinks alginate and TA by reacting with the hydroxyl groups, introducing intermolecular bridges between the polysaccharide chains. Here, the more numerous struts within the intra-lamellar hollow spaces and their evolution into a honeycomb-like structure substantially enhanced the robustness and stability of the GTA-crosslinked aerogels. This substantially enhanced all the reported mechanical parameters. However, the short chain length of GTA restricted the free molecular motion of both alginate and TA, resulting in increased brittleness. For instance, A5C5T2-X* reached a 30% strain before undergoing failure and compaction at higher strains. Notwithstanding, alternative crosslinking agents such as carboxylic acid or genipin may yield higher toughness but lower compressive strength [31].

### 2.6. Thermal Transport Properties

The effective conductivity of a foam-like material (Equation (4)) can be estimated by summing the contributions of several mechanisms: conduction through the solid phase ($\lambda_{s}$), conduction through the gas phase ($\lambda_{g}$), radiation through the structure ($\lambda_{r}$), and heat convection through the cells ($\lambda_{c}$). This last term can be neglected when the pore size is smaller than 2 mm [32].

(4) $$\lambda =\lambda_{s}+\lambda_{g}+\lambda_{r}+\lambda_{c}$$

The solid phase conductivity ($\lambda_{s}$) is determined by the phonon transmission through the aerogel’s solid skeleton; its contribution can be approximated by the following expression:

(5) $$\lambda_{s}=\lambda_{0}f\frac{\rho_{s}}{\rho_{b}}$$

where $\rho_{s}$ is the skeletal density, $\rho_{b}$ is the bulk density, and $\lambda_{0}$ is the solid conductivity. The efficiency factor (f) accounts for the characteristics of the porous structure, such as tortuosity, hole geometry, cross-section, and structure continuity; it takes the value of 1/3 for open-pore structures [33]. The conductivity of a dense solid ($\lambda_{0}$) can be calculated by averaging the contribution of each component via the rule of mixtures. The thermal conductivities of neat alginate, TA, and clay were 0.454, 0.415, and 0.951 W/m·K, respectively; And the thermal conductivity of the gas phase corresponds to the air inside the aerogel ($\lambda_{air}$ = 0.026 W/m·K).

(6) $$\lambda_{g}=\lambda_{air}\left(1-\frac{\rho_{s}}{\rho_{b}}\right)$$

**Figure 7:**
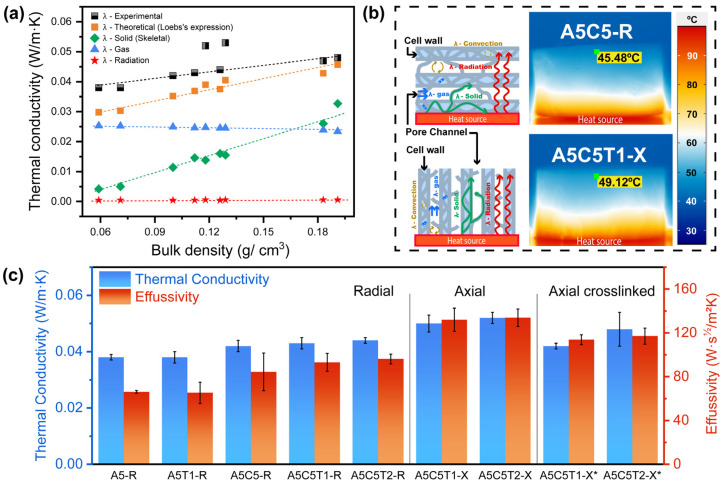
(**a**) Correlation between the thermal conductivities and bulk densities of the AA aerogel composites, (**b**) Scheme of different contributions in thermal conductivity and thermography of A5C5-R and A5C5T1-X aerogels on a hot plate surface at 100 °C, and (**c**) Thermal conductivity and effusivity of AA composite aerogels.

The contribution of radiation decreases with the increase in density and pore size. In the present case and at room temperature, it was considered negligible [34]. Nevertheless, it was estimated using Loeb’s expression, which considers the thermal radiation conductivity across the pores [35].

(7) $$\lambda_{r}=4\varepsilon \gamma \sigma dT^{3}$$

where ε is the emissivity of the sample (ε=0.9), σ is the Stefan–Boltzmann constant, d is the average pore size in the heat-flow direction (~100 μm) and T is the temperature of the sample. The expression γ=π/4 applied for cylindrical pores perpendicular to the heat flow, and γ=1 applied if they were aligned with the heat flow. Figure 7a illustrates the different contributions and the measured global thermal conductivities.

The radial-aligned alginate aerogel composites showed experimental thermal conductivities in the range of 0.038–0.044 W/m·K (Figure 7c and Table S4), in line with those previously reported in the literature [2,11]. Pure alginate exhibited the lowest thermal conductivity (0.031 W/m·K), which increased as the clay content increased. This outcome was expected, as clay is a higher thermal conductor than the alginate matrix (λ=0.951 W/m·K) [36]. The axially oriented aerogels showed the highest thermal conductivities, as expected, with A5C5T1-X and A5C5T2-X exhibiting the highest values. This result was attributed to the convection term, which had a non-negligible effect in this case. Assuming cells going only across half of the aerogel monolith, the Grashof number calculation yielded a value of 3050 (β=1/298; ∆T=2 °C; L=12.5 mm; $\rho_{air}=$1.176 Kg/m^3^; $\mu_{air}=$2 × 10^−5^ Ns/m^2^) [37]. This value is approximately three times higher than the limit (1000) to activate the convection term.

In addition, the conductivity of the axial crosslinked specimens decreased compared to their non-crosslinked counterparts, showing values similar to the sum of the solid and gas conduction contributions. Both crosslinking and the higher viscosity of the precursor solutions created additional links and struts bridging the columnar pores, resulting in a notable decrease in the effective pore size in the heat-flow direction (Figure 7a). Reducing the pore size below 2 mm made convection negligible. On the other hand, the increase in density and the number of struts led to the opening of new heat-flow paths, resulting in a proportional increase in heat conduction through the solid skeleton.

The thermal effusivity substantially increased in the axial non-crosslinked and crosslinked samples, indicating a greater ability to interchange heat with their surroundings (Figure 7c). However, the thermal diffusivity decreased in parallel to the porosity and the increase in solids. Pure alginate reached its thermal equilibrium the fastest, whereas longer times were needed for axial non-crosslinked and crosslinked samples.

The thermal diffusivity of the aerogels decreased considerably with increasing relative density, that is, with an increasing volume fraction of solids (Vs). The relationship between the thermal diffusivity and the fraction of solids can be expressed as follows:

(8) $$\alpha =K{V_{s}}^{-\beta }$$

where K is a constant, and β indicates the dependency between both parameters and reflects the pore structure. The value of β for the radial and crosslinked samples was 0.56, similar to that of other aerogels (0.5–0.6) [38]. The lowest diffusivity values corresponded to the axially oriented aerogels.

To further study the thermal insulation performance of aerogels, we monitored the evolution of the upper face aerogel’s temperature with time, under constant heating at 100 °C (Videos S4 and S5). The curves are shown in Figure S5. Scheme of the different thermal conductivity contributions in the radially and axially oriented aerogel, as well as the infrared thermographic images of representative aerogels (A5C5T1-R and A5C5T1-X*) were also taken (Figure 7b) after 20 min of heating. As a result, A5C5T1-R showed the highest thermal insulation capacity, indicating greater difficulty transferring heat compared to its axial counterpart. Although small differences in temperature were observed between the samples, the temperature from bottom to top decreased by nearly 50% in all cases, indicating that the prepared aerogels had adequate insulation capacity.

### 2.7. Thermal Stability

The thermal properties of the AA composite aerogels were analyzed using thermogravimetric analysis (TGA) and derivative thermogravimetry (DTG) under an N_2_ atmosphere. Table 3 lists the onset of decomposition at 5% mass loss after water evaporation (T*_onset_*), the maximum decomposition temperature (Td*_max_*), the mass decomposition rate (dW/dT*_max_*), and the residue percentage at 600 °C.

**Figure 8:**
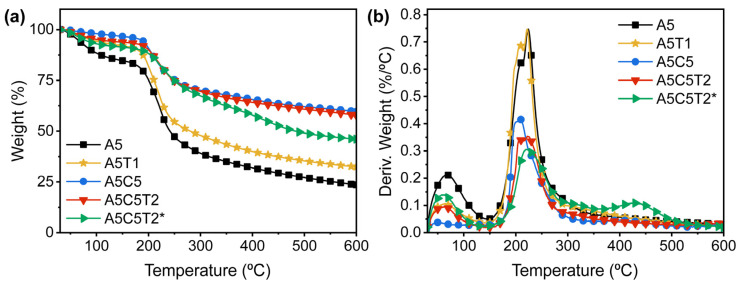
(**a**) TGA weight loss; (**b**) derivative thermogravimetric curves of alginate composite aerogels.

Aerogel composites presented an initial weight loss derived from physisorbed water loss. The pristine alginate aerogel (A5) exhibited the greatest weight loss between 150 and 400 °C, caused by the dehydroxylation, decarboxylation, and breakage of glycosidic linkages, releasing H_2_O, CO_2_, and CO (Figure 8a) [39]. The rate of mass decomposition was 0.74 %/°C, leaving a residue of 23.6%. A displacement of the Td*_max_* from 224 to 222 °C was observed when TA was incorporated into the alginate matrix (A5T1). Subsequently, the outer galloyl moieties decomposed in the range of ~225 to 400 °C, forming 1,2,3-benzenetriol and CO_2_. Moreover, the 37% increase in the residue was attributed to the decomposition of inner galloyl units and the formation of crosslinked aromatic structures (Figure 8b) [40].

The clay’s positive effect on thermal stability was evident in A5C5. T*_onset_* increased by 3% because of the dominant diffusion barrier effect of clay that limits oxygen access into the polymer. Moreover, the presence of 3d transition metal cations (Fe and Cu) in MMT clay promotes the radical trapping mechanism, delaying the depolymerization of alginate [41]. Compared to TA, the clay decelerated the dW/dT*_max_* by 43% between 200 and 300 °C and increased the char residue ~2.5-fold.

A two-step degradation curve was observed for GTA-crosslinked aerogels. The Td*_max_* did not change, but a new peak appeared around 430 °C, which may be due to a possible cyclization mechanism. The most remarkable effect of GTA was on the deceleration of the degradation rate, which was lowered to 0.28%/°C for A5C5T1-X* and 0.31%/°C for A5C5T2-X*. The acetalization reaction reduced the molecular chain mobility and therefore increased the thermal stability of the crosslinked aerogels.

### 2.8. Fire Behavior and Char Mechanism

Given the possible applications of the developed aerogels, it is worth understanding their fire behavior and the role of each component in fire prevention. Hence, bench-scale cone calorimetry was performed, and Raman spectroscopy was used to assess the ashes after total oxygen combustion. Cone calorimetry is based on the oxygen consumption principle, which measures the heat generated per unit of oxygen consumed. Therefore, the heat release rate (HRR) is one of the most critical fire reaction properties, as it directly influences other parameters, such as the time to ignition (TTI), the peak of heat release (PHRR), the time to peak heat release (TTPHRR), the total heat release (THR), and the total effective heat release (THReff). All these parameters are listed in Table 4.

The heat release rate (HRR) is determined by analyzing the oxygen content in the fuel gases. This process is based on the principle that the heat released from a fuel is directly related to the oxygen consumed during combustion. The average heat of combustion for most materials is approximately 13.1 MJ/kg of oxygen [42]. As a result, the cone calorimeter software calculates the HRR by evaluating the mole fraction of O_2_ in the exhaust gas and comparing it to the O_2_ concentration in the air (20.95%) [43].

**Figure 9:**
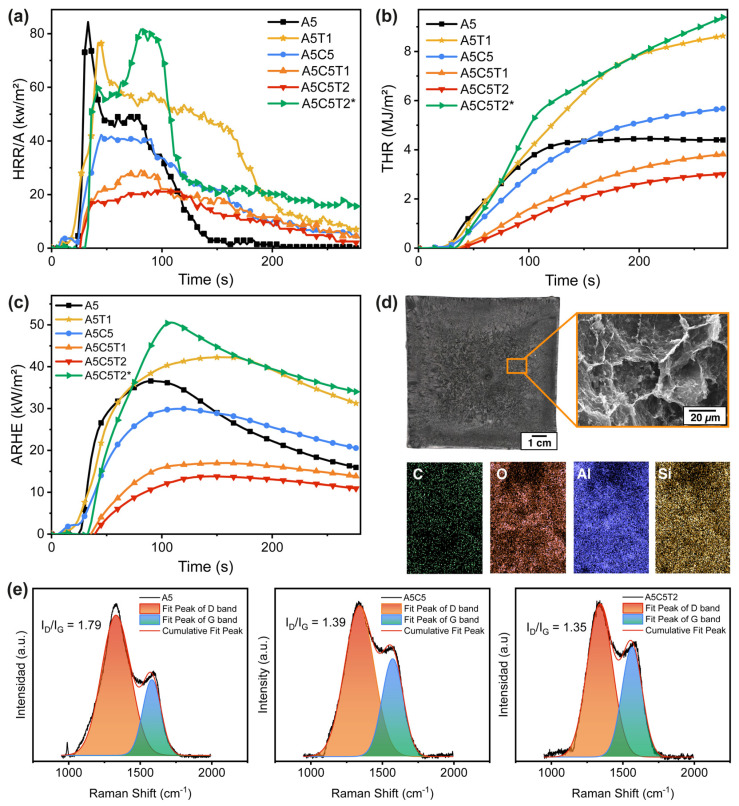
Main representative (**a**) HRR, (**b**) THR, and (**c**) ARHE curves from ammonium alginate composites; (**d**) photograph and SEM photomicrograph and EDS elemental mapping of the char of A5C5T2 after cone calorimetry; (**e**) Raman spectra of A5, A5C5, and A5C5T2 aerogel ashes.

The neat AA aerogel ignited after just 11 s and showed a sharp flame at 33 s, with a low PHRR of 84.4 kW/m^2^, indicating low fire intensity (Figure 9a). This initial flame resulted from the heat released during alginate dehydration (the loss of physically adsorbed and chemically bound water), following the decarboxylation and esterification of alginate chains, while releasing NH_4_^+^ ions and H_2_O, CO_2_, and CO [44]. A wide shoulder appeared between 50 and 100 s as a result of char generation through the combustion of these gases. Finally, as the char was oxidized and smoldered, the fire intensity and fume release decreased. The PHRR values aligned with the PHHR of 71.27 kW/m^2^ reported by Shang et al. [45]. However, substantial differences in the TTI were observed, probably due to the variation in the nitrogen content and its sources.

The signal of A5T1 was similar to that of pure alginate, suggesting that the sample absorbed the radiation from the cone calorimeter, reaching a similar concentration of volatile gases, although the process took longer because of the physical interaction between AA and TA and the higher activation energy of TA [40]. Thus, TA is effective as a charring agent only when protected from direct oxygen action [46]. Therefore, TA caused an increment in the THR (Figure 9b).

By contrast, the A5C5 aerogel did not ignite, reducing the PHRR by half (42 kW/m^2^) and the THReff (0.92 MJ/m^2^·g). These improvements were due to the protective effect of the laminated clay. Logically, the amount of residue increased notably because of the inorganic nature of clay, which blocks the heat and mass transfer by losing only its crystal structure and retaining its structural integrity, while alginate is combusted [45]. Likewise, when AA was combined with both TA and MMT clay, a synergistic effect was observed, resulting in a further reduction of the fire parameters, with the A5C5T2 sample presenting the lowest PHRR (21.1 kW/m^2^) and THReff (0.34 MJ/m^2^·g). This corroborates the excellent thermal stability and fire-protective qualities of these materials (Video S6).

However, a rapid rise in HRR was observed (Figure 9a) when aerogels were crosslinked with GTA because the flash point of this compound occurs at a lower temperature (79 °C). Thus, the PHRR was increased up to 2.8-fold and THReff up to 2.6-fold compared to their non-crosslinked counterparts. Thus, while GTA enhanced the mechanical properties, it did not contribute to the fire resistance of the aerogels.

Additional parameters, such as the fire growth index (FIGRA, defined as the ratio between PHRR and TTPHRR), the average rate of heat emission (ARHE), and the fire performance index (FPI, defined as the ratio between TTI and PHRR), were also evaluated. A5C5T2 showed the lowest FIGRA value, and its FPI could not be determined because of its non-burning behavior. Therefore, this sample demonstrated the lowest potential fire risk. Furthermore, this sample showed the lowest maximum average heat rate (MARHE), demonstrating its ability to suppress flames and prevent their propagation (Figure 9c).

The heating process in the cone calorimetry test primarily involves unidirectional forced heat transfer from the heater to the sample through radiation. During heat exposure, the material’s ability to absorb radiant energy becomes a critical factor as the sample temperature rises. Although there are also radiation and convection losses from the top surface as the material heats up, these losses are less important, implying that absorptivity is more critical than emissivity. In the high-temperature environment of the cone calorimeter, heat transfer is mainly accomplished by thermal radiation. In our case, the wavelength of thermal radiation was concentrated around 2.5 μm. Therefore, the material’s resistance to fire may be increased using conformal high-reflective and low-absorptivity coatings containing nanoparticles such as SiC, SiO_2_, or TiO_2_, which act as an excellent infrared shielding layer [47]. Furthermore, adding metal hydroxides results in dehydration, lowering the temperature of both the condensed phase and combustible gas. Future developments may also include using metamaterials as thermal cloaks to protect the samples from heat [48].

The char residue was analyzed through Raman spectroscopy and scanning electron microscopy (SEM). Figure 9e depicts the Raman spectra of the A5, A5C5, and A5C5T2 aerogel composites. The spectra showed two peaks: a D peak in the 1325–1335 cm^−1^ range and a G peak in the 1582–1593 cm^−1^ range. The D peak was associated with the disordered graphitic carbon, whereas the G peak was attributed to the in-plane crystalline graphite carbon vibrations. The relative intensity ratio between the D and G bands (ID/IG) determines the graphitization degree and the number of surface defects of carbon materials, where a lower ID/IG means a better graphite structure. The ID/IG ratio of pure alginate was 1.79. The A5C5 sample presented a much lower value of ID/IG = 1.39. Finally, the ID/IG ratio of A5C5T2 was 1.35. These values are below those reported for AA/phytic acid aerogels [49], evidencing that the MMT clay exerts a predominant effect in the graphitization of the ashes, resulting in a better-condensed phase mechanism.

The photograph and SEM photomicrograph in Figure 9d show the resultant char of A5C5T2, evidencing the structural integrity of the aerogel after combustion. No holes or cracks were present, and a continuous and homogeneous layer was formed on the top aerogel surface. On the other hand, the EDS analysis revealed that the main elements of char (C, O, Na, Mg, Al, and Zn) were well dispersed along the entire surface.

**Figure 10:**
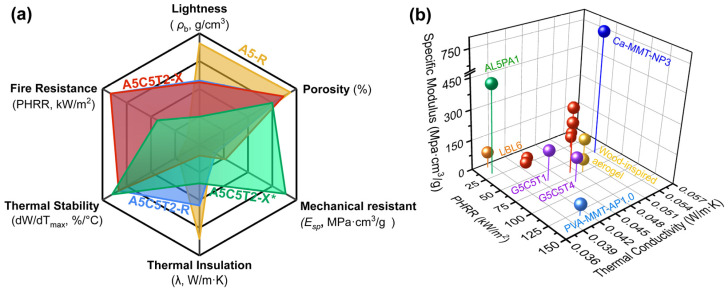
(**a**) Comparison of the compositions and properties of the aerogels evaluated in this study; (**b**) comparison of our ammonium alginate aerogels with other aerogel composites reported in the literature [49,50,51,52,53,54].

To contextualize the performance of the aerogels developed in this study, Figure 10a depicts a general overview of the properties of the aerogels developed in this study, along with a comparative scheme of the thermal conductivity, fire-resistance, and mechanical properties of bio-based aerogel composites reported in the literature (Figure 10b) [49,50,51,52,53,54]. As illustrated in the figure, the aerogels developed in this study demonstrated significantly higher values than those observed in aerogel composites based on cellulose filaments reinforced by layer by layer deposition of chitosan/phytic acid/citric acid (LBL6) [51], axially oriented wood-inspired aerogel crosslinked with Ca^2+^/H_3_BO_3_ [52], PVA/Aramid pulp reinforced with MMT (PVA-MMT-AP1.0) [53], and gelatin modified with MMT clay and TA (G5C5T1 and G5C5T4) [54]. Regarding thermal insulation and fire resistance, our aerogels are also competitive with the tubular cellulose aerogel composite reinforced with clay (Ca-MMT-NP3), as reported by Sun et al. [50]. However, their performance is slightly lower compared to the AA aerogel reinforced with attapulgite and crosslinked with phytic acid (AL5PA1) developed by Cao et al. [49] Despite this, in a general view, the AA aerogels composites developed exhibit competitive performance compared to these similar natural-based systems.

## 3. Conclusions

High-performance multifunctional bio-based aerogels with radial and axial structural orientation were obtained by freeze-casting. The combination of ammonium alginate (AA) as the polymer matrix and the montmorillonite (MMT) clay/tannic acid (TA) reinforcements provided the aerogels with increased rigidity corresponding to the increment in the solid content. Similarly, arranging the ice template following the ice-growth direction enhanced the mechanical performance. The highest compressive resistance was achieved when the arrangement was axial (A5C5T2-X), reaching a 3.6-fold increase (Esp = 57.1 MPa·cm^3^/g) compared with the radially oriented sample (Esp = 15.8 MPa·cm^3^/g).

Furthermore, incorporating clay within the aerogel structures substantially improved their thermal stability and flame resistance and suppressed combustion because of clay’s barrier effect. Combining Si, Al, and Mg from MMT clay with phenol moieties from TA further reduced heat emission by up to 50% (PHRR = 21.1 kW/m^2^) for the A5C5T2 aerogel. Furthermore, Raman spectroscopy revealed that this combination improved the graphitization efficiency and fire safety of the aerogels in high-temperature environments.

Furthermore, glutaraldehyde (GTA) crosslinking played a crucial role in generating acetal bridges, which increased the struts/linkages and therefore the tortuosity among the axial pore channels, reinforcing the honeycomb-like structure observed in the scanning electron microscope (SEM) photomicrographs. This structural arrangement substantially improved the insulation properties of the aerogels and reduced the thermal conductivity to 0.048 W/m·K compared to the non-crosslinked axial counterpart. The acetalization of alginate enhanced the mechanical behavior of the A5C5T2-X* sample 5.3-fold (305.29 MPa·cm^3^/g) compared to the non-crosslinked samples. However, the flame resistance of the GTA-crosslinked aerogel decreased up to 2.8-fold (PHRR = 67.4 kW/m^2^) because of the earlier flash point of GTA. Despite this, the properties of the ammonium alginate aerogel composites are comparable to or better than those of the aerogels reported in the literature, representing a step forward in the development of alternatives to petroleum-based foams.

## 4. Materials and Methods

### 4.1. Materials

Ammonium alginate (AA) (Cecalgum A 500, 350–450 mPa·s) with a mannuronic to guluronic acid ratio of 1.37 was purchased from Algaia (Paris, France). Tannic acid (TA) powder and glutaraldehyde (GTA) solution (50 wt.% in H_2_O) were obtained from Merck (Barcelona, Spain). Sodium montmorillonite (MMT) clay (PGW grade, ρ = 2.6 g/cm^3^) was obtained from Nanocor (Hoffman Estates, IL, USA).

### 4.2. Aerogel Preparation

Figure 1 depicts the preparation process of the aerogel composites. Briefly, 5 g of AA and 5 g of MMT clay were dispersed separately in 40 mL of deionized (DI) water by mechanical stirring. TA powder was dissolved in 20 mL of DI water and added dropwise to the alginate solution while continuously stirring at 200 rpm. The MMT solution was then added to the TA–alginate mixture, and the system was homogenized for 20 min at 9000 rpm using an IKA T−25 Ultra-Turrax disperser (Staufen, Germany). The final formulations were poured into 30 mm diameter cylindrical and square-shaped (100 × 100 × ~10 mm) molds.

The radial orientation during freeze casting was attained by immersing the cylindrical samples into a dry ice/ethanol bath (−80 °C) while keeping the bath level at the same height as the solution in the mold. On the other hand, the axial orientation was achieved by placing the molds on a copper plate that was in contact with liquid nitrogen (−196 °C), allowing freezing from the base to the top of the mold.

In situ crosslinking was achieved by incorporating 10 mL of GTA solution into the TA–alginate blend and stirring at 200 rpm for 40 min. After homogenization, the MMT clay solution was added to the blend and stirred for another 20 min at 9000 rpm, using the IKA T-25 Ultra-Turrax disperser. The mixtures were poured into the previously mentioned molds, frozen to attain the axial direction, and lyophilized.

### 4.3. Characterization

The AA’s molecular weight was analyzed through intrinsic viscosity measurements using a Ubbelohde Type 1B glass capillary viscometer and a VB-1423 precision viscosimeter bath (J.P. Selecta, Barcelona, Spain) at 25 °C, in accordance with ASTM D2857.

To analyze the chemical structure of the aerogels, a Nicolet 6700 Fourier Transform Infrared (FTIR) spectrophotometer (Thermo Fisher Scientific, Waltham, MA, USA) set in attenuated total reflectance (ATR) mode was used. A resolution of 1 cm^−1^ over the 4000 and 400 cm^−1^ range was adopted.

The chemical composition of the crosslinked aerogels was analyzed using X-ray photoelectron spectroscopy (XPS). XPS spectra were obtained using a D8 Advance system (SPECS Surface Nano Analysis Gmbh, Berlin, Germany) equipped with a PHOIBOS 150 EP hemispherical energy analyzer and an MCD-9 detector, using an Al Kα X-ray source with an energy of 1486.6 eV and power of 100 W and a pass energy of 20 eV. The setup was positioned at 54° with respect to the analyzer axis and calibrated by the 3d5/2 line of Ag with a full width at half maximum (FWHM) of 1.211 eV. A flood gun operating at an emission of 13 μA and 1.5 eV was used. Survey spectra were obtained with an energy step of 1 eV, and high-resolution spectra were acquired with an energy step of 0.1 eV.

The aerogels’ bulk density ($\rho_{b}$) was determined by the ratio of weight to volume of cylindrical monoliths (d = 30 mm, h = 25 mm). The dimensions of the sample were measured using a digital vernier caliper (Mitutoyo CD-20DC, Kawasaki, Japan), and the weight was recorded using an analytical balance (Shimadzu AUW120D, Kyoto, Japan). Five replicates were taken for each composition. Samples were previously stabilized under standard atmosphere conditions (T = 25 °C, RH = 50%). The total water content in the stabilized samples was determined using an HE53 moisture-loss analyzer (Mettler Toledo, Greifensee, Switzerland) after 30 min of exposure at 160 °C.

Relative density ($\rho_{r}$), describing the solid fraction of the aerogels, was calculated as the ratio of the $\rho_{b}$ to the skeletal density ($\rho_{s}$) and measured using a helium pycnometer (Micromeritics AccuPyc 1330, Norcross, GA, USA). The aerogel porosity was determined using the following equation:

(9) $$P=\left(1-\rho_{r}\right)\cdot 100$$

The Brunauer–Emmett–Teller (BET) specific surface area ($S_{BET}$) and the Barret–Joyner–Halenda (BJH) pore size distribution of representative aerogels were examined by nitrogen adsorption/desorption isotherms measured at 77.3 K using a Micromeritics system (Norcross, GA, USA).

The morphology of the aerogels and the clay element dispersion analysis were observed with a scanning electron microscope (SEM) (Jeol 001F, Tokyo, Japan) equipped with an energy-dispersive X-ray spectrometer (EDS). Samples were cryo-fractured and made conductive by deposition of 80% platinum and 20% palladium alloy. Three-dimensional-structure computerized images were performed by X-ray microtomography (µ-CT) using a Skyscann 1272 MP (Bruker, MA, USA) with a source voltage of 40 kV, current of 200 µA, exposure time of 1.5 s, and an image pixel size of 0.8 μm.

The mechanical properties of the aerogels were evaluated in compression mode using a universal testing machine (ZwickRoell Z010, Ulm, Germany) fitted with a 10 kN load cell and tested at a crosshead speed of 1 mm/min. A minimum of five replicates were tested.

The thermal conductivity (λ) and effusivity (e) of the aerogels were measured using a hot disk analyzer on the modified transient plane source (TPS) method (C-Therm, TCi Thermal Conductivity Analyzer, Fredericton, NB, Canada), using a power level of 60 mW, test time of 2.7 s, and cooling time of 90 s. A minimum of five samples were averaged. The thermal conductivity of pure substances, namely AA, TA, and clay, was determined by measuring the powders compacted as a disc (d = 40 mm and h = 3 mm). Thermal grease (Wakefield type 120 silicone) was used as a contact agent between the disc and the sensor. The diffusivity (α), defined as the rate of heat transfer within a material from the hot side to the cold side, was calculated from the specific heat capacity ($C_{p}$) formula as follows:

(10) $$C_{p}=\frac{e^{2}}{\lambda \rho_{b}}$$

(11) $$\alpha =\frac{\lambda }{{\rho_{b}C}_{p}}$$

The thermal dynamic distribution and temperature of aerogels on a heating plate were recorded using an infrared (IR) camera (InfraTec ImageIR 800, Dresden, Germany) equipped with a mercury–cadmium–telluride (MCT) detector. The temperature resolution at 30 °C was higher than 0.035 K. The IRBIS 3.1 professional software was used to collect the temperatures along the sample’s longitudinal axis.

A Mettler Toledo TGA thermogravimetric analyzer (Mettler Toledo, Greifensee, Switzerland) was used to determine the material’s thermal stability. An approximately 10 mg sample was heated at 10 °C/min from 30 °C to 600 °C under an N_2_ atmosphere. Three replicates per composition were tested.

Combustion behavior was assessed using an Ineltec BECC cone calorimeter (Barcelona, Spain) following ISO 5660, under a heat flux of 50 kW·m^−2^. The samples dimensions were 100 × 100 × ~10 mm. The char residues were analyzed by Raman spectroscopy performed on a Renishaw’inVia Qontor microscope (England, UK) with an infrared laser at 785 nm laser (power 1%).

## Figures and Tables

**Table 1:** XPS surface atomic compositions and ratios.

Sample	C 1S (%)	N 1S (%)	O 1S (%)	O/C
A5	60.945	2.292	36.763	0.603
A5*	68.145	2.651	29.204	0.428
A5C5T2*	63.448	1.714	34.838	0.549

**Table 2:** Mechanical performance of the aerogel composites.

Sample	E (MPa)	E_sp_ (MPa·cm^3^/g)	σy (MPa)	σy_sp_ (MPa·cm^3^/g)	E_abs_ at 50% Strain (kJ/m^3^)
A5-R	0.03 ± 0.01	0.52 ± 0.12	-	-	0.58 ± 0.11
A5T1-R	0.06 ± 0.04	0.84 ± 0.56	-	-	0.72 ± 0.08
A5C5-R	0.96 ± 0.21	9.53 ± 2.09	0.05 ± 0.01	0.49 ± 0.04	5.09 ± 0.15
A5C5T1-R	1.58 ± 0.50	14.08 ± 4.45	0.09 ± 0.01	0.81 ± 0.02	8.11 ± 0.80
A5C5T2-R	1.99 ± 0.50	15.79 ± 4.03	0.11 ± 0.01	0.87 ± 0.07	9.12 ± 0.63
A5C5T1-X	6.66 ± 1.28	54.60 ± 5.42	0.38 ± 0.02	3.17 ± 0.34	21.81 ± 0.99
A5C5T2-X	7.57 ± 1.69	57.10 ± 6.40	0.44 ± 0.05	3.34 ± 0.05	23.79 ± 1.88
A5C5T1-X*	47.06 ± 11.53	254.76 ± 51.89	1.06 ± 0.31	5.75 ± 1.46	57.49 ± 1.41
A5C5T2-X*	58.28 ± 8.41	305.29 ± 46.05	1.34 ± 0.03	7.04 ± 0.22	64.02 ± 2.33

**Table 3:** Thermogravimetric characteristics of composite aerogels.

Sample	T*_onset_* (°C)	Td*_max_* (°C)	dW/dT*_max_* (%/°C)	W_R_ (%)
A5	189	224	0.74	23.6
A5T1	183	222	0.75	32.4
A5C5	195	209	0.42	59.6
A5C5T1	200	209	0.36	57.6
A5C5T2	200	222	0.35	57.8
A5C5T1*	201	218	0.28	50.3
A5C5T2*	203	220	0.31	46.1

**Table 4:** Results of cone calorimetry.

Sample	TTI (s)	PHRR (kW/m^2^)	TTPHRR (s)	THR ^♦^ (MJ/m^2^)	THReff ^∇^ (MJ/m^2^·g)	FPI (kW/m^2^·s)	FIGRA (s·m^2^/kW)	MARHE (kW/m^2^)	W_R_ (%)
A5	11	84.4	33	4.0	1.07	0.13	2.56	36.17	1.48
A5T1	21	76.4	42	8.6	1.81	0.27	1.82	42.26	1.51
A5C5	No Flame	42.3	45	5.7	0.92	-	0.94	29.96	42.69
A5C5T1	No Flame	27.7	69	3.8	0.41	-	0.40	16.99	43.26
A5C5T2	No Flame	21.1	87	3.0	0.34	-	0.24	13.79	39.64
A5C5T1*	6	67.4	39	7.7	0.85	0.09	1.73	47.97	34.35
A5C5T2*	8	59.7	42	9.3	0.89	0.13	1.42	50.58	23.96

^♦^ at 276 s; **^∇^** after total oxygen consumption.

## Data Availability

All data are available on request from the corresponding author. The data are not publicly available due to ongoing researches using a part of the data.

## Supplementary Materials

The following supporting information can be downloaded at: https://www.mdpi.com/article/10.3390/gels10080526/s1. Table S1: Aerogel compositions; Table S2: Densities, porosity, and water content of aerogel compositions; Table S3: Main FTIR bands presented in AA aerogel composites; Table S4: Thermal insulation properties of aerogel composites. Figure S1: Proposed chemical reactions of precursors; Figure S2. (a) FTIR spectra of all aerogel compositions, and (b) GTA-crosslinked FTIR-signal; Figure S3. The wide energy scan spectra (WESS) of A5, A5*, A5C5T2* aerogel compositions; Figure S4. Stabilization curve of AA aerogel composites; Figure S5. The evolution of the upper face aerogel’s temperature with time under constant heating at 100 °C. Videos S1–S6 are available at https://www.mdpi.com/article/10.3390/gels10080526/s1. Videos S1 and S3: µ-CT 3D view of A5-R, A5-X, and A5C5T2* aerogels architecture, respectively; Videos S4 and S5: Thermographic evolution of A5C5T1-R and A5C5T1-X aerogel on a hot plate surface, respectively. Videos S6: Flame-resistant behavior of A5C5T2 aerogel under 50 kW·m^−2^ by cone calorimetry.
